# Alexithymic traits and dissociative episodes in borderline patients

**DOI:** 10.1192/j.eurpsy.2025.1918

**Published:** 2025-08-26

**Authors:** S. Costantini, L. Ghidetti, M. Benassi, M. Pacetti, D. Salmaso, R. P. Sant’Angelo

**Affiliations:** 1 Mental Health, Ausl Romagna; 2 Psychology, University of Bologna, Cesena; 3 Mental Health Department, Ausl Romagna, Forlì; 4 Mental Health Department, Ausl Romagna, Cesena, Italy

## Abstract

**Introduction:**

The biopsychosocial theory (Paris, 1996) considers the complex interaction of biological, psychological, and social factors in the development and manifestation of borderline personality disorder. Among the environmental factors are unstable family relationships, unresolved traumatic experiences, and adverse life events that can foster alexithymia and dissociative defense mechanisms, as supported by several studies in the literature (Carano et al., 2011; Caretti et al., 2005; Macrì et al., 2022).

**Objectives:**

The present study aims to investigate these constructs in a population of borderline patients admitted to the Psychiatry Department at Bufalini Hospital in Cesena.

**Methods:**

The sample was selected using the SCID PD (Structured Clinical Interview for DSM-5), followed by the administration of the following scales: TAS-20 (Toronto Alexithymia Scale), DES II (Dissociative Experiences Scale), SHI (Self-Harm Inventory), and BSQ (Body Shape Questionnaire). Multivariate analyses were applied, including non-parametric correlations (Spearman’s Rho) between variables using SPSS software.

**Results:**

The sample consisted of 20 individuals (F = 17; M = 3) with a mean age of 27.57 years (SD = 7.83). Regarding the TAS, 85% of the participants reported clinically significant scores (Tot >50) compared to the healthy adult population, whose Italian mean is 44. For the DES, 70% of the sample reached clinically significant scores (Tot >30), whereas the healthy adult population scores below 10.

The data revealed a statistically significant positive correlation between high TAS-20 and DES II scores, indicating a relationship between alexithymia and dissociative symptoms. Furthermore, while a trend toward correlation was observed between TAS-20 and BSQ, as well as DES II and BSQ, and SHI and BSQ, these correlations were not statistically significant.

**Conclusions:**

In line with previous literature, most individuals with borderline personality disorder exhibit alexithymia (85%) and experience dissociative episodes (70%). The present findings demonstrate a significant relationship between alexithymia and dissociative symptoms, highlighting how individuals with greater difficulty recognizing and describing their emotions tend to report more frequent episodes of depersonalization and derealization. This suggests that those with such difficulties may use dissociation as a defensive strategy to avoid intense emotions and stressful situations. Additionally, it would be valuable to further explore the relationship between body satisfaction, dissociative symptoms, alexithymia, and self-harm, as these manifestations may be linked to emotional processing difficulties. These findings contribute to a better understanding of emotional and dissociative dynamics in patients with borderline personality disorder and underscore the importance of integrated assessment during treatment.

**Disclosure of Interest:**

None Declared

